# Subcortical structural changes along the menstrual cycle: beyond the hippocampus

**DOI:** 10.1038/s41598-018-34247-4

**Published:** 2018-10-30

**Authors:** Belinda Pletzer, TiAnni Harris, Esmeralda Hidalgo-Lopez

**Affiliations:** Department of Psychology & Centre for Cognitive Neuroscience, University of Salzburg, Salzburg, Austria

## Abstract

Animal studies have robustly shown hormone related changes in spine density in various brain areas, specifically the hippocampus. Literature on hormone dependent gray matter volume changes in humans is however less consistent. While various areas have been reported to change along the menstrual cycle in women, many do not survive multiple-comparisons correction and only hippocampal changes have been replicated. We attribute these problems to small sample sizes and inconsistent definitions of menstrual cycle phases. In the present study a large sample of 55 women was scanned three times along their menstrual cycle in concisely defined time windows of hormonal changes. Accordingly this is the first study using a large enough sample size to assess menstrual cycle dependent changes in human brain structure with sufficient power. Results confirm a significant estradiol-dependent pre-ovulatory increase in gray matter volumes of the bilateral hippocampus, but also show a significant, progesterone-dependent increase in gray matter volumes of the right basal ganglia after ovulation. No other areas were affect by hormonal changes along the menstrual cycle. These hormone driven menstrual cycle changes in human brain structure are small, but may be the underlying cause of menstrual cycle dependent changes in cognition and emotion.

## Introduction

In animal studies it is well established that steroid hormones, including the sex hormones estradiol and progesterone, affect brain structure. Specifically, estradiol increases spine density in the rat hippocampus^1^ and prefrontal cortex^2^. However, only few studies have tried to confirm these results in humans using functional magnetic resonance imaging. These studies focused either on gray matter volumes as obtained with voxel-based morphometry^3–8^ or on cortical thickness^9^. They generally use rather small sample sizes and differ with respect to study design (longitudinal vs. cross-sectional, group-based vs. single-subject), the cycle phases included (e.g. menses vs. pre-menstrual; pre-ovulatory vs. peri-ovulatory) and the definition and assessment of menstrual cycle phases (e.g. self-reports vs. hormone-analyses). Accordingly, results are hard to compare between the different studies, but some consensus arises regarding the hippocampus as will be detailed in the following.

First results on the topic were reported by Protopopescu *et al*.^3^ on 21 naturally cycling women, including both, women with (11) and without (10) pre-menstrual symptoms. They compared women in their post-menstrual/pre-ovulatory phase (days 10–12 post-menstruation) and pre-menstrual phase (days 1–5 prior to menstruation). They were indeed able to find larger gray matter volumes in the right hippocampus of women during their pre-ovulatory cycle phase (high estradiol, low progesterone) compared to their pre-menstrual cycle phase (falling estradiol and progesterone levels), while the right basal ganglia (putamen/pallidum) showed smaller gray matter volumes during the pre-ovulatory compared to the pre-menstrual phase. Exploratory analyses did not survive multiple-comparisons correction but suggested larger volumes in the left middle frontal gyrus (MFG) during the pre-ovulatory and larger volumes in the left superior parietal lobule and right anterior cingulate cortex (ACC) during the pre-menstrual phase. However, the definition of cycle phases in this study was rather lenient and was not confirmed by hormone analyses. Specifically, the pre-menstrual cycle phase included a rather large window during which hormone levels may still be high. Nevertheless, their result of hippocampal structural changes across the menstrual cycle was confirmed by Linsofsky *et al*.^7^ as well as a recent longitudinal single-subject study^8^. Across four menstrual cycles of the same subject, the pre-ovulatory peak in estradiol was paralleled by a peak in bilateral hippocampal gray matter volumes and fractional anisotropy as assessed with diffusion tensor imaging. Note however, that this study – while including multiple days of the menstrual cycle across several menstrual cycles – was performed on only a single subject.

In 2010 a study on only 14 women^4^ found slightly larger gray matter volumes in the right parahippocampal/fusiform gyrus of women during their early follicular phase (low estradiol and progesterone) compared to their mid-luteal cycle phase (high estradiol and progesterone). This study did however not include the pre-ovulatory cycle phase, which is characterized by peak levels of estradiol only. Follow-up studies on 24 women found larger regional gray matter volumes during the pre-ovulatory phase compared to the luteal phase in the right ACC, the right MFG and the left insula^5,6^. While the results in the MFG are comparable to the results reported by^3^ albeit in a different hemisphere, the results in the ACC are opposite to those reported by^3^. However, only the results in the left insula did actually survive correction for multiple comparisons^6^. These results are probably most similar to results obtained by a recent study on cortical thickness^9^. They found significantly smaller cortical thickness values during the luteal phase compared to the early follicular phase in the lateral orbitofrontal cortex. Note however, that this was a between-subjects study focused mostly on hormonal contraceptive effects, which did not correct for multiple comparisons.

In summary, previous research in humans suggests the hippocampus, basal ganglia and insula as potential targets of structural changes in response to sex hormone fluctuations across the menstrual cycle, with some trend findings in the parahippocampal/fusiform gyrus, ACC and middle frontal gyrus. However, different studies reach different conclusions, which is attributable (i) to the inclusion of different cycle phases and (ii) rather small sample sizes. While the results in humans suggest, that results from animal studies can be observed in humans using magnetic resonance imaging, systematic studies including all three cycle phases and sufficiently large sample sizes are lacking. This is particularly problematic since neuroimaging studies, which base their analyses on thousands of voxels, often suffer from insufficient power^10^. In the present study we seek to overcome these problems, by pooling structural imaging data of women from two fMRI studies on the menstrual cycle conducted on the same scanner using the same T1-weighted sequence and strict criteria for the definition of menstrual cycle phases. In a first step, we seek to replicate previously reported structural changes via region of interest based (ROI) analyses. We hypothesize to confirm an increase in hippocampal volume during the pre-ovulatory cycle phase compared to menses, as well as an increase in grey matter volumes of the basal ganglia, MFG and Insula in the luteal phase compared to menses. In a second step, we seek to explore structural changes across the menstrual cycle in other brain areas via whole-brain analyses.

## Results

**Figure 1:**
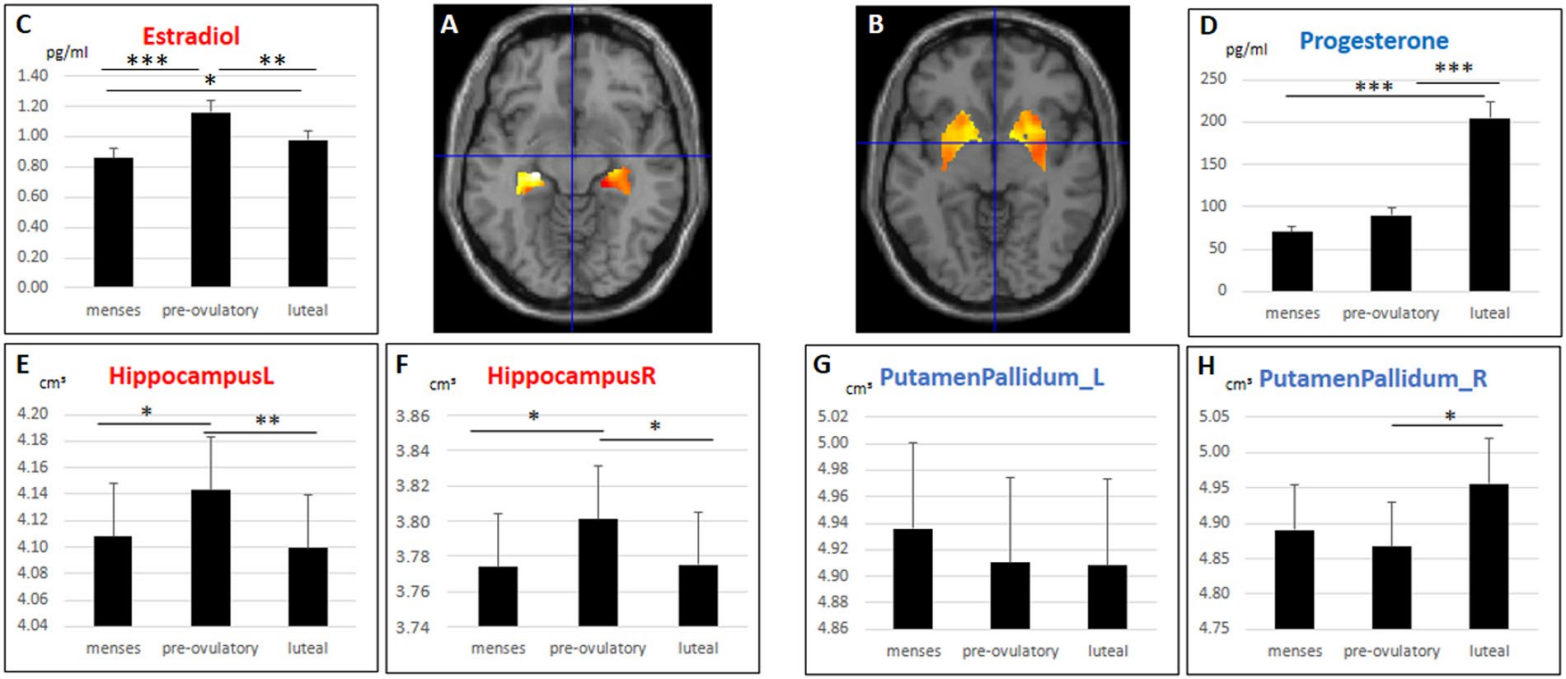
Subcortical structural changes along the menstrual cycle. Gray matter volumes extracted from the left and right hippocampus were significantly larger in the pre-ovulatory phase, when estradiol levels peak, compared to menses and luteal cycle phase. The effect was indeed driven by estradiol levels. Gray matter volumes extracted from the right basal ganglia were significantly larger in the luteal phase, when progesterone levels peak, compared to the pre-ovulatory phase. The effect was indeed driven by progesterone levels. (**A**) Hippocampal regions of interest, (**B**) Basal ganglia regions of interest, (**C**) Estradiol levels during the three cycle phases, (**D**) Progesterone levels during the three cycle phases, (**E,F**) Left and right hippocampal volumes during the three cycle phases, (**G,H**): left and right basal ganglia volumes during the three cycle phases. ^*^p < 0.05, ^**^p < 0.01, ^***^p < 0.001. Error bars represent standard errors.

Results are summarized in Fig. 1.

### Hormone analyses

Estradiol (Fig. 1C) was significantly higher during the pre-ovulatory phase compared to menses (b = 0.59, SE_b_ = 0.11, t_(108)_ = 5.62, p < 0.001), as well as during the luteal phase compared to menses (b = 0.24, SE_b_ = 0.11, t_(108)_ = 2.25, p = 0.03). Furthermore, estradiol was significantly higher during the pre-ovulatory phase compared to the luteal phase (b = −0.35, SE_b_ = 0.12, t_(54)_ = −2.79, p = 0.007). Progesterone (Fig. 1D) was significantly higher during the luteal phase compared to menses (b = 1.20, SE_b_ = 0.12, t_(108)_ = 9.78, p < 0.001) and the pre-ovulatory phase (b = 0.90, SE_b_ = 0.12, t_(54)_ = 7.48, p < 0.001), but did not differ between menses and pre-ovulatory phase (b = 0.20, SE_b_ = 0.12, t_(108)_ = 1.61, p = 0.11).

### ROI analyses

#### Hippocampus

Both, left and right hippocampal gray matter volumes (Fig. 1E,F) were significantly increased during the pre-ovulatory cycle phase compared to menses (left: b = 0.09, SE_b = _0.03, t_(107)_ = 3.07, p = 0.003, p_FDR_ = 0.02, right: b = 0.09, SE_b_ = 0.03, t_(107)_ = 2.62, p = 0.01, p_FDR_ = 0.04) and the luteal cycle phase (left: b = −0.11, SE_b_ = 0.03, t_(53)_ = 3.58, p < 0.001, p_FDR_ = 0.006, right: b = −0.08, SE_b_ = 0.03, t_(53)_ = −2.66, p = 0.01, p_FDR_ = 0.04), with no differences between luteal phase and menses (both b > 0.02, both SE_b_ = 0.03, both t_(107)_ < 0.72, both p > 0.47). This effect was indeed driven by estradiol level as indicated by a small, but significant correlation between estradiol and hippocampal GM volumes in both hemispheres (left: b = 0.05, SE_b_ = 0.022, t_(108)_ = 2.32, p = 0.02, right: b = 0.06, SE_b_ = 0.03, t_(108)_ = 2.19, p = 0.03; results not shown).

#### BG (Putamen/Pallidum)

No menstrual cycle dependent changes were observed in the left BG (all b > 0.05, all SE_b_ > 0.07, all t_(107)_ > 0.82, all p > 0.41; Fig. 1G). Gray matter volumes in the right BG (Fig. 1H) increased significantly during the luteal phase compared to the pre-ovulatory phase (b = 0.18, SE_b_ = 0.07, t_(53)_ = 2.41, p = 0.02, p_FDR_ = 0.05), with no differences between the other cycle phases (both b < 0.13, both SE_b_ = 0.08, both t_(107)_ < 1.51, both p > 0.13). This effect was driven by progesterone levels as indicated by a significant correlation between progesterone and right BG volumes (b = 0.10, SE_b_ = 0.04, t_(108)_ = 2.15, p = 0.03; results not shown), while no significant effect of estradiol was observed (b = 0.02, SE_b_ = 0.06, t_(108)_ = 0.36, p = 0.72).

#### Insula

GM volumes in the Insula were not modulated by menstrual cycle phase (all b < 0.06, all SE_b_ = 0.03, all t < 1.79, all p > 0.075; results not shown).

#### MFG

Gray matter volumes in the left MFG were smaller during the mid-luteal cycle phase compared to menses (b = −0.10, SE_b_ = 0.05, t_(107)_ = −2.01, p = 0.046, p_FDR_ = 0.32; results not shown). However, the effect did not survive correction for multiple comparisons. There was no differences between the other cycle phases (all b < 0.05, all SE_b_ > 0.04, all t < 1.00, all p > 0.32) and no menstrual cycle effects were observed on right MFG volumes (all b < 0.07, all SE_b_ > 0.04, all t < 1.37, all p > 0.17). Left MFG volumes were not related to either estradiol (b = −0.01, SE_b_ = 0.04, t_(108)_ = −0.32, p = 0.74) or progesterone levels (b = −0.01, SE_b_ = 0.02, t_(108)_ = −0.53, p = 0.59).

### Whole brain analyses

In the whole brain analyses no other area was modulated by menstrual cycle phase or sex hormone levels (results not shown). Also the areas showing significant results in the ROI analyses did not survive multiple-comparisons correction in the whole-brain analyses.

## Discussion

The present study aimed at investigating menstrual cycle dependent changes in brain areas that had previously emerged as potential targets for sex hormone fluctuations including the hippocampus^1,3,7,8^, the BG (putamen/pallidum)^3^, the Insula^6^ and the MFG^2,3,5,6,11^. Furthermore, we sought to explore whether hormonal fluctuations along the menstrual cycle affected any other brain area outside these ROIs.

We found an estradiol-dependent increase in gray matter volumes in the hippocampus during the pre-ovulatory phase of the menstrual cycle. This effect is consistent with previous findings^3,7,8^. Note that the hippocampus emerged as the only estradiol-sensitive area with respect to gray matter volumes in this study. This effect has previously been linked to cognitive changes along the menstrual cycle^3^.

Furthermore we observed a progesterone-dependent increase in gray matter volumes in the right BG during the luteal phase of the menstrual cycle. This effect has also been previously described^3^, but has not yet been related to progesterone levels before. Importantly, this effect was lateralized to the right hemisphere, while no menstrual cycle dependent changes were observed in the left BG. Since the changes in the right BG show the largest effect size, we speculate that these changes may be related to the emotional changes, which have been consistently reported after ovulation, specifically during the late luteal/premenstrual cycle phase, when estradiol and progesterone levels are falling^11^. Reduced basal ganglia volumes, specifically of the right basal ganglia, have been observed in mood disorders like unipolar and bipolar depression^12^ and have been linked to associated depressive symptoms in neurological disorders like Parkinsons disease^13^. Accordingly, a reduction in right basal ganglia volume after the mid-luteal progesterone peak might mediate hormone-related mood swings observed in the second half of the luteal cycle phase prior to the onset of menses.

No menstrual cycle dependent changes were observed beyond these regions of interest. Specifically, none of the previously reported effects in cortical areas, like the Insula or middle frontal gyrus, could be confirmed. Thus hormone-dependent changes in gray matter volumes along the menstrual cycle in the present study were restricted to subcortical areas, with distinct targets of estradiol and progesterone. However, we want to point out that the effect size of these changes is rather small. The largest effect size was observed in the right BG. Thus these effects might be easier to detect in smaller subcortical areas rather than large cortical gyri like the middle frontal gyrus. If only a specific part of the middle frontal gyrus is target by sex hormones, these changes may not show up when extracting volumes from the whole area. The voxel-based whole-brain analysis is more sensitive to such localized changes, but requires a stricter control for multiple comparisons, which may mask small effects. This low sensitivity in detecting small cortical changes represents a limitation of the current study. However, since cortical effects have not been reported with consistency before, it is also possible that previous results represent false positives due to small sample sizes and/or insufficient control of menstrual cycle phases.

The large sample size and very strict criteria for inclusion of menstrual cycle phases along with hormone assessment represent particular strength of the current study. In fact, this is the first menstrual cycle study of brain structure using a large enough sample size to assess these effects with sufficient power. In summary, our results corroborate those menstrual cycle-dependent effects which had already been reported with consistency before.

## Material and Methods

### Participants

78 healthy young women aged 18–35 years, were recruited for one of two different fMRI-studies. Of these women, 55 (mean age: 25.67 years, SD = 4.34 years) were included in the final analysis (see Procedure). Participants had no psychological, neurological or endocrinological disorders according to self-reports and they did not display any brain tissue abnormalities on the structural MRI. Only participants who had not been using any hormonal contraceptives or IUDs for the past 6 months were included in the study. All participants had a regular menstrual cycle of 21–35 days in length (mean cycle length: 28.13 days, SD = 2.35 days). Informed written consent was obtained from participants in both studies. Both studies were approved by the University of Salzburg’s ethics committee and conform to the Code of Ethics of the World Medical Association (Declaration of Helsinki).

### Procedure

In both studies, participants completed 3 scanning sessions time-locked to the early follicular, pre-ovulatory and mid-luteal cycle phase. In order to schedule the scanning sessions, cycle duration was calculated based on participants self-reports of the dates of onset of their last three periods. Early follicular scans were carried out between the second and fifth day of their menstrual cycle. In order to schedule pre-ovulatory scans, expected ovulation was calculated as 14 days before the expected onset of next menses and confirmed by commercial urinary ovulation tests (Pregnafix®). These tests indicate a rise in Luteinizing hormone (LH) 3–4 days before ovulation, while the estradiol peak is expected 2–3 days before ovulation. Accordingly, the pre-ovulatory session was scheduled 2–3 days before the expected ovulation and was carried out only, if commercial ovulation tests showed their first positive result on the day of testing or the day before. The mid-luteal session was scheduled earliest 3 days after ovulation and up to three days before the expected onset of next menses. Cycle phases were additionally confirmed by onset of next menses on the one hand and salivary hormone levels (compare 2.6.) on the other hand. Of the 78 participants, 8 did not complete structural scans during all three sessions and 15 were excluded due to inconsistencies between calculated cycle phases and hormone values as described in section 4.5. These participants were excluded from the analyses. For the remaining 55 participants, the menses session on average took place on day 3.67 (SD = 1.47), the pre-ovulatory session on average took place on day 12.05 (SD = 2.44), the luteal session on average took place on day 21.78 (SD = 4.24).

Although not of relevance for the structural images, the order of cycle phases across scanning sessions was counter-balanced in order to avoid learning effects on the task-based scans acquired during the same session. 16 women had their first session during menses, 19 women during their pre-ovulatory phase and 20 women during their luteal phase.

### MRI data acquisition

High resolution structural images were acquired on a Siemens Magnetom TIM Trio 3 Tesla scanner (Siemens Healthcare). We use a T1-weighted 3D MPRAGE sequence (160 sagital slices, slice thickness = 1 mm, TE 291 ms, TR 2300 ms, TI delay 900 ms, FA 9°, FOV 256 × 256 mm). In both studies, the high resolution structural scan was acquired as fourth scan after a fieldmap, a resting state functional scan, and a task-based functional scan of 20–30 minutes length, whereby the task varies between the two studies.

### MRI data analysis

For data analysis of the structural data, we used the voxel-based morphometry (VBM) approach as implemented in the CAT12 toolbox (http://dbm.neuro.uni-jena.de/vbm/) of the SPM12 software (http://www.fil.ion.ucl.ac.uk/spm/). During VBM, structural images are spatially registered to an anatomical template, tissue in each voxel is classified as grey matter, white matter or CSF, bias correction is applied to control for intensity non-uniformities. Furthermore, segmentation are modulated by the volume change due to spatial registration^14^. CAT12 provides an option for longitudinal segmentation using each subject as his/her own template during spatial registration. Accordingly, this option is more sensitive to small volumetric changes within the same subject. The default options for CAT12 longitudinal segmentation were used. These include the use of SPM12 tissue probability maps and European brain templates for affine regularization during the initial SPM12 affine registration, as well as light affine preprocessing and moderate (0.5) strength of local adaptive segmentation, skull stripping and final clean-up for CAT12 longitudinal segmentation. Intra-subject bias was corrected using spatial normalization to the same stereotactic space (MNI template) and voxel size for normalized images was set to 1.5 mm. The different brain segments were modulated using nonlinear normalization parameters to control for individual differences in brain size.

In a first step, gray matter volumes were extracted using the get_totals script by G. Ridgeway (http://www0.cs.ucl.ac.uk/staff/gridgway/vbm/get_totals.m) from the following regions of interest, for which menstrual cycle dependent effects have previously been described to survive multiple comparisons correction: (i) the left and right hippocampus (Fig. 1A), which emerged as areas showing an estradiol-dependent pre-ovulatory increase in GM volumes in multiple studies, (ii) the left and right basal ganglia (putamen/pallidum; Fig. 1B), since the right putamen/pallidum emerged as area showing a pre-menstrual increase in GM volumes in^3^ (iii) the left and right Insula, since the right Insula emerged as area showing a significant menstrual cycle effect in^6^. In addition, gray matter volumes were extracted from the left and right MFG, because even though this region did not survive multiple comparisons correction in human menstrual cycle studies, it was reliably modulated by sex hormone levels on animal studies^2,15^. Aal-masks to extract these volumes were created using the wfu-pickatlas. The extracted volumes were compared between cycle phases using linear mixed effects models in R 3.4.0 (compare Statistical analyses).

In a second step, whole-brain comparisons were performed using SPM12 second level analysis, in order to see, whether menstrual cycle affected any area outside these regions of interest.

Before entering SPM12 second level analysis, the gray matter segments were smoothed using an 8 mm full width at half maximum (FWHM) Gaussian kernel. The smoothed gray matter segments were then subjected to a within-subjects ANOVA with the factor cycle-phase in SPM12. Contrasts comparing each cycle phase to each other were defined and a peak-level FDR-corrected threshold was used.

### Hormone analysis

In each study saliva samples were collected from participants via the passive drool method before and after entering the scanner. In the first study only one saliva sample was collected before and after scanning (i.e. two samples in total), in the second study two saliva samples were collected before and after scanning (i.e. four saliva samples in total). Saliva samples were stored at −20 °C until hormone assessment and were centrifuged twice, for 15 min and 10 min, respectively at 3000 rpm. Prior to analysis, the samples for each session were be pooled, in order to assess an averaged value over the course of the scanning session. In both studies, estradiol was assessed using Salimetrics High Sensitivity salivary estradiol assays and progesterone was assessed using DeMediTec Progesterone free in saliva ELISAs. Both assays provide ELISA plates á 96 wells. Subtracting the wells needed for standards and controls and taking into account the fact that all samples were analyzed in duplicates to account for errors in pipetting, samples of 13 subjects (3 sessions per subject) were analyzed on one ELISA plate. Accordingly, samples of one subject were always analyzed on the same plate, but multiple plates were needed for each study. The average of duplicate values was used for statistical analyses. If the coefficient of variation between duplicates exceeded 25%, the analysis was repeated for all samples of that subject. Observed hormone values were within the expected range for the respective assays. On the one hand, hormone values were used to confirm cycle phases as calculated based on self-reports and ovulation tests. Participants were expected to show the highest estradiol levels during the pre-ovulatory phase and the highest progesterone levels during the luteal phase. Due to individual variation and methodological restrictions this was of course not expected to apply in all participants. However, participants were excluded if they showed their lowest estradiol levels during the pre-ovulatory phase (9 participants) or their lowest progesterone levels during the luteal phase (5 participants) or both (1 participant). Two samples differing by less than the assay sensitivity of 0.1 pg/ml for estradiol and 5 pg/ml for progesterone were considered comparably high. On the other hand, hormone values were used as covariates in the above mentioned analyses to clarify, whether estradiol or progesterone are responsible for any menstrual cycle changes observed.

### Statistical analyses

Statistical analyses were performed in R 3.4.0 using linear mixed effects models. Participant number was modelled as a random factor. For comparison of hormone levels between cycle phases, only cycle phase was entered as a fixed effect (formula: hormone ~ 1|PNr + cycle). For the comparison of GM volumes between cycle phases, total intracranial volume (TIV) and age were modeled as covariates of no interest (formula: GM ~ 1|PNr + cycle + TIV + age). In all analyses, a first model included all participants and cycle phases (165 observations) in order to compare the high-hormone phases (pre-ovulatory and luteal phase) to menses. A second model was then run excluding menses (110 observations) to compare pre-ovulatory and luteal phase to each other. P-values were FDR-corrected to control for multiple-comparisons (eight ROIs in total). If a significant menstrual cycle change was observed in the pre-ovulatory phase, the cycle factor was replaced by estradiol to confirm that the effect was indeed caused by an increase in estradiol. If a significant menstrual cycle change was observed in the luteal phase, the cycle factor was replaced by estradiol and progesterone respectively to evaluate which hormone drove the effect. The data file and script used for statistical analyses is openly available at http://webapps.ccns.sbg.ac.at/OpenData/. MR images are available upon request from the first author.
